# Interferons and HIV Infection: The Good, the Bad, and the Ugly

**DOI:** 10.20411/pai.v1i1.125

**Published:** 2016-07-16

**Authors:** Netanya S. Utay, Daniel C. Douek

**Affiliations:** 1 Division of Infectious Diseases, Department of Medicine, University of Texas Medical Branch, Galveston, Texas; 2 Human Immunology Section, Vaccine Research Center, National Institutes of Allergy and Infectious Diseases, National Institutes of Health, Bethesda, Maryland

**Keywords:** HIV, SIV, AIDS, interferon type I, IFN, CD4

## Abstract

Whether type I interferons (IFNs) hinder or facilitate HIV disease progression is controversial. Type I IFNs induce the production of restriction factors that protect against mucosal HIV/SIV acquisition and limit virus replication once systemic infection is established. However, type I IFNs also increase systemic immune activation, a predictor of poor CD4^+^ T-cell recovery and progression to AIDS, and facilitate production and recruitment of target CD4^+^ T cells. In addition, type I IFNs induce CD4^+^ T-cell apoptosis and limit antigen-specific CD4^+^ and CD8^+^ T-cell responses. The outcomes of type I IFN signaling may depend on the timing of IFN-stimulated gene upregulation relative to HIV exposure and infection, local versus systemic type I IFN-stimulated gene expression, and the subtype of type I IFN evaluated. To date, most interventional studies have evaluated IFNα2 administration largely in chronic HIV infection, and few have evaluated the effects on tissues or the HIV reservoir. Thus, whether the effect of type I IFN signaling on HIV disease is good, bad, or so complicated as to be ugly remains a topic of hot debate.

**STANDFIRST**

Type I interferons simultaneously control HIV replication and induce systemic immune activation, but whether the net effect is beneficial or detrimental remains controversial.

Type I interferons (IFNs) include 13 IFNα subtypes, IFNβ, IFNε, IFNκ and IFNω. All type I IFNs bind the cell surface receptor IFNAR, a heterodimer of IFNAR1 and IFNAR2, but with varying affinities for each subunit [1, 2]. Binding and signaling through IFNAR results in the transcription of a multitude of IFN-stimulated genes (ISGs), and while all type I IFNs can activate antiviral pathways, the specific ISGs transcribed may vary widely [2].

Plasma IFNα levels are increased within the first week of HIV infection [3]. Based on data from rhesus macaque studies, ISGs are upregulated in peripheral blood and lymphoid tissues during acute simian immunodeficiency virus (SIV) infection, and despite decreasing, ISG expression remains higher in chronic SIV infection compared to pre-infection levels [4–6]. While continued ISG expression in chronic HIV infection may reflect the host's efforts to control virus replication and delay progression to AIDS, ISG expression has also been proposed to contribute directly to increasing the systemic inflammation that has been implicated in disease progression and mortality. Indeed, the complex system of IFN signaling renders classifying its biological outcome as good, bad or even ugly, somewhat of a challenge.

## THE GOOD …

Type I IFNs stimulate expression of HIV restriction factors that limit virus replication. These restriction factors act at virtually every stage of the HIV replicative cycle, from reverse transcription (SAMHD1 and APOBEC3) to nuclear entry (MX2) to transcription (Schlafen 11) and budding (tetherin) [7–12]. Endogenous type I IFN production likely contributes some protection against infection, consistent with data that transmitted/founder viruses are relatively resistant to type I IFNs [13, 14]. Intramuscular IFNα2a administration to rhesus macaques prevented systemic SIV infection following intrarectal inoculation as long as ISGs, including restriction factors, were upregulated [6]. In addition to upregulation in peripheral blood mononuclear cells, restriction factors were upregulated in rectal tissue and lymph nodes, protecting against transmission across the mucosal barrier and against dissemination, respectively. Similarly, vaginal IFNβ administration prevented simian/human immunodeficiency virus (SHIV) infection in rhesus macaques following vaginal challenge with concomitant upregulation of ISGs in vaginal suspensions [15], suggesting that expression of antiviral proteins at the site of inoculation is sufficient to protect against systemic infection. Clearly, therefore, type I IFN signaling can prevent retrovirus infection in primates.

Type I IFN signaling may also be beneficial during acute retrovirus infection. Decreasing or delaying type I IFN signaling by *in vivo* blockade of IFNAR during acute SIV infection increased the SIV RNA setpoint [6], suggesting that the precise timing of ISG expression determines host control of virus production. Administration of IFNα2 during chronic HIV infection tends to decrease HIV RNA and p24 antigen levels but is by no means a panacea as it has yet to be shown that it improves clinical outcomes beyond antiretroviral therapy (ART) alone [7, 16–27]. However, recent data from *in vitro* and humanized mice studies suggest that IFNα8 and IFNα14 suppress HIV replication to a much larger extent than IFNα2 [28, 29], likely reflecting their higher affinities for IFNAR and subsequent increased MX2 and tetherin expression and APOBEC3 activity. Thus, type I IFN signaling can both prevent retrovirus infection and suppress retrovirus replication after infection.

## THE BAD …

Clearly the elaboration of type I IFNs constitutes a proinflammatory response. In HIV infection, increased systemic immune activation predicts poor CD4^+^ T-cell recovery on ART and increased morbidity and mortality [30, 31]. Many have postulated that natural hosts of SIV such as sooty mangabeys and African green monkeys do not progress to AIDS because they downregulate ISGs and systemic immune activation just weeks after SIV infection [4, 5]. Notably, these species suffer less immunologic and structural damage to the gut during acute infection, which may explain the decreased inflammation in chronic infection[32]. Thus, once chronic SIV infection is established, these natural hosts have reverted to pre-infection levels of immune activation.

Several mechanisms have been proposed to explain this association of immune activation with disease progression. Increased ISG expression in CD4^+^ T cells is associated with CD4^+^ T-cell depletion [33], possibly via apoptosis mediated by tumor necrosis factor (TNF)-related apoptosis-inducing ligand (TRAIL) [34]. Type I IFNs also upregulate the HIV coreceptor CCR5 and induce pDC production of CCR5 ligands, creating and recruiting more target cells and further facilitating CD4^+^ T-cell depletion [35, 36]. In addition, type I IFNs suppress thymic output, further limiting CD4^+^ T-cell recovery [37]. Indeed, higher circulating IFNα levels are associated with lower CD4^+^ T cell counts [38], and IFNα2a administration decreases CD4^+^ T cell counts in HIV-infected people [39]. Thus, while type I IFNs can suppress virus replication, they are also associated with increased CD4^+^ T-cell depletion.

In addition, chronic type I IFN signaling suppresses adaptive immunity. In the LCMV mouse model, blockade of type I IFN signaling improved antigen-specific CD4^+^ T-cell responses [40, 41], so type I IFNs not only suppress CD4^+^ T-cell recovery but also functionality. Type I IFNs do stimulate CD8^+^ T-cell activation and proliferation [27, 38, 42]. However, if type I IFN signaling precedes antigen exposure, proliferation of antigen-specific CD8^+^ T cells is suppressed [43]. Thus, HIV-infected people exposed to new antigens, including in the context of vaccination, may have suboptimal T-cell responses. Together, these findings suggest that chronic type I IFN signaling can increase susceptibility of HIV-infected persons to other infections.

## THE UGLY …

Whether HIV clinical outcomes can be improved by increasing or decreasing type I IFN signaling remains hotly debated. The effect of type I IFN signaling on HIV disease pathogenesis varies based on whether acquisition, acute infection, chronic untreated infection, or chronic infection with virologic suppression is considered.

Although systemic IFNα2a prevented SIV acquisition after rectal challenge [6], the findings may be different with vaginal challenge. The rectum is rich in CCR5^+^ CD4^+^ T cells, which may be readily infected with SIV [44]. In contrast, in the endocervix, pDCs are recruited to the site of infection where type I IFNs induce pDC production of CCR5 ligands that recruit CD4^+^ T cells. As a result, HIV-infected clusters form that serve as a nidus for dissemination [36]. While these pDCs may produce type I IFNs and induce antiviral protein expression, infection also spreads along the path of these infiltrating inflammatory cells [36]. Topical glycerol monolaureate was shown to suppress chemokines that recruit pDCs and prevent dissemination [36]. Paradoxically, topical IFNβ also prevented infection despite increasing the frequency of CCR5^+^ CD4^+^ T cells, likely due to simultaneous upregulation of antiviral mediators [15]. By accessing visceral tissues and lymphatic tissues, systemic type I IFN administration may facilitate proliferation, recruitment, and activation of pDCs and CD4^+^ T cells and thereby facilitate dissemination or, alternatively, prevent spread with widespread induction of antiviral genes. The specific restriction factors critical for HIV control are also unknown, and an approach focused specifically on increasing the host's ability to limit HIV replication rather than upregulating all IFN signaling may result in an intervention with greater tolerability and fewer adverse consequences.

The precise timing of type I IFN signaling in acute retrovirus infection is critical for determining clinical outcomes. Insufficient IFN signaling in the first week of infection can result in rapid progression to AIDS [6]. However, whether boosting type I IFN signaling during acute infection can limit the reservoir has yet to be determined. Data from African green monkeys suggest no adverse consequences from IFNα2 administration starting 9 days post-infection, although ISGs were not further upregulated [45]. Rhesus macaques treated with type I IFN chimeras during the first 3 months of SIV infection had a similar disease course to untreated animals, but the impact on IFN signaling was not evaluated [46]. Thus, whether IFN signaling can be further induced in acute retrovirus infection remains unknown. Certainly it is tempting to speculate that increased and prolonged restriction factor expression may limit the HIV reservoir and even result in long-term control.

In chronic untreated infection, the data are fairly consistent that IFNα2a can suppress HIV [7]. However, with the efficacy and tolerability of current antiviral regimens, the most clinically relevant question is whether type I IFNs can impact residual virus that persists despite ART. One potential limitation of antiretroviral medications is their ability to achieve sufficiently high levels in tissues such as lymph nodes [47]. Type I IFNs readily penetrate lymph nodes, the gastrointestinal tract, central nervous system, and other tissues [48]. Nonetheless, in people with chronic HIV infection taking suppressive ART, IFNα2 treatment has not had a dramatic impact on CD4 T-cell recovery to date, nor has it had a clear detrimental impact [7]. However, these studies have not comprehensively evaluated the impact of type I IFNs on HIV levels in tissues. All studies published to date in chronic infection in non-human primates and humans have used IFNα2, so whether IFNα8 and IFNα14 would have a more pronounced effect is unknown. In addition, repetitive IFNα2a inoculation can eventually cause an IFN-tolerant state, which increased the virus burden in our rhesus macaque study [6], but type I IFNs with stronger IFNAR affinity that induce different ISGs may not carry the same risk.

The interaction of HIV and type I IFNs is complicated. Factors such as the anatomical site of HIV exposure or stage of HIV disease may determine whether type I IFN signaling is beneficial or detrimental. The 17 different type I IFNs each bind to their receptor differently and induce different signaling cascades, yet most studies in humans have only evaluated one of these type I IFNs. Several questions remain:

1. Can boosting type I IFN signaling during acute HIV/SIV infection result in a smaller reservoir and slower disease progression?
2. Does systemic type I IFN administration protect against or predispose to vaginal acquisition of HIV/SIV?
3. Are type I IFNs other than IFNα2 more effective *in vivo* at suppressing HIV/SIV replication during acute and chronic infection? Are they more pro-inflammatory or immunosuppressive?
4. Can IFN-induced restriction factors be upregulated in a focused way without risking the potential detrimental consequences of boosting systemic type I IFN signaling?
5. Would blocking IFN signaling in chronic infection contribute to a reduction in systemic immune activation and thus ameliorate clinical outcome?

Ultimately, much remains to be explored to determine whether the functionality of type I IFNs can be harnessed to prevent or cure HIV infection, and we would encourage further clinical studies in humans.

## References

[1] Schreiber G, Piehler J Te molecular basis for functional plasticity in type I interferon signaling. Trends Immunol. 2015;36(3):139–49. PubMed PMID: . doi: 10.1016/j.it.2015.01.002 25687684

[2] Gibbert K, Schlaak JF, Yang D, Dittmer U IFN-alpha subtypes: distinct biological activities in anti-viral therapy. Br J Pharmacol. 2013;168(5):1048–58. PubMed PMID: Pubmed Central PMCID: . doi: 10.1111/bph.12010 23072338 PMC3594665

[3] Stacey AR, Norris PJ, Qin L, Haygreen EA, Taylor E, Heitman J, Lebedeva M, DeCamp A, Li D, Grove D, Self SG, Borrow P Induction of a striking systemic cytokine cascade prior to peak viremia in acute human immunodeficiency virus type 1 infection, in contrast to more modest and delayed responses in acute hepatitis B and C virus infections. J Virol. 2009;83(8):3719–33. PubMed PMID: Pubmed Central PMCID: . doi: 10.1128/jvi.01844-08 19176632 PMC2663284

[4] Bosinger SE, Li Q, Gordon SN, Klatt NR, Duan L, Xu L, Francella N, Sidahmed A, Smith AJ, Cramer EM, Zeng M, Masopust D, Carlis JV, Ran L, Vanderford TH, Paiardini M, Isett RB, Baldwin DA, Else JG, Staprans SI, Silvestri G, Haase AT, Kelvin DJ Global genomic analysis reveals rapid control of a robust innate response in SIV-infected sooty mangabeys. J Clin Invest. 2009;119(12):3556–72. PubMed PMID: . 19959874 10.1172/JCI40115 PMC2786806

[5] Jacquelin B, Mayau V, Targat B, Liovat AS, Kunkel D, Petitjean G, Dillies MA, Roques P, Butor C, Silvestri G, Giavedoni LD, Lebon P, Barre-Sinoussi F, Benecke A, Muller-Trutwin MC Nonpathogenic SIV infection of African green monkeys induces a strong but rapidly controlled type I IFN response. J Clin Invest. 2009;119(12):3544–55. PubMed PMID: . 19959873 10.1172/JCI40093 PMC2786805

[6] Sandler NG, Bosinger SE, Estes JD, Zhu RT, Tarp GK, Boritz E, Levin D, Wijeyesing-he S, Makamdop KN, del Prete GQ, Hill BJ, Timmer JK, Reiss E, Yarden G, Darko S, Contijoch E, Todd JP, Silvestri G, Nason M, Norgren RB Jr., Keele BF, Rao S, Langer JA, Lifson JD, Schreiber G, Douek DC Type I interferon responses in rhesus macaques prevent SIV infection and slow disease progression. Nature. 2014;511(7511):601–5. PubMed PMID: Pubmed Central PMCID: . doi: 10.1038/na-ture13554 25043006 PMC4418221

[7] Bosinger SE, Utay NS Type I interferon: understanding its role in HIV pathogenesis and therapy. Curr HIV/AIDS Rep. 2015;12(1):41–53. PubMed PMID: . doi: 10.1007/s11904-014-0244-6 25662992

[8] Schoggins JW, Wilson SJ, Panis M, Murphy MY, Jones CT, Bieniasz P, Rice CM A diverse range of gene products are effectors of the type I interferon antiviral response. Nature. 2011;472(7344):481–5. PubMed PMID: . 21478870 10.1038/nature09907 PMC3409588

[9] Pillai SK, Abdel-Mohsen M, Guatelli J, Skasko M, Monto A, Fujimoto K, Yukl S, Greene WC, Kovari H, Rauch A, Fellay J, Battegay M, Hirschel B, Witteck A, Berna-sconi E, Ledergerber B, Gunthard HF, Wong JK Role of retroviral restriction factors in the interferon-alpha-mediated suppression of HIV-1 in vivo. Proc Natl Acad Sci U S A. 2012;109(8):3035–40. PubMed PMID: . 22315404 10.1073/pnas.1111573109 PMC3286922

[10] Goujon C, Moncorge O, Bauby H, Doyle T, Ward CC, Schaller T, Hue S, Barclay WS, Schulz R, Malim MH Human MX2 is an interferon-induced post-entry inhibitor of HIV-1 infection. Nature. 2013;502(7472):559–62. PubMed PMID: . 24048477 10.1038/nature12542 PMC3808269

[11] Kane M, Yadav SS, Bitzegeio J, Kutluay SB, Zang T, Wilson SJ, Schoggins JW, Rice CM, Yamashita M, Hatziioannou T, Bieniasz PD MX2 is an interferon-induced inhibitor of HIV-1 infection. Nature. 2013;502(7472):563–6. PubMed PMID: . 24121441 10.1038/nature12653 PMC3912734

[12] Schoggins JW, MacDuf DA, Imanaka N, Gainey MD, Shrestha B, Eitson JL, Mar KB, Richardson RB, Ratushny AV, Litvak V, Dabelic R, Manicassamy B, Aitchison JD, Aderem A, Elliott RM, Garcia-Sastre A, Racaniello V, Snijder EJ, Yokoyama WM, Diamond MS, Virgin HW, Rice CM Pan-viral specificity of IFN-induced genes reveals new roles for cGAS in innate immunity. Nature. 2013;505(7485):691–5. PubMed PMID: . 24284630 10.1038/nature12862 PMC4077721

[13] Parrish NF, Gao F, Li H, Giorgi EE, Barbian HJ, Parrish EH, Zajic L, Iyer SS, Decker JM, Kumar A, Hora B, Berg A, Cai F, Hopper J, Denny TN, Ding H, Ochsenbauer C, Kappes JC, Galimidi RP, West AP Jr., Bjorkman PJ, Wilen CB, Doms RW, O'Brien M, Bhardwaj N, Borrow P, Haynes BF, Muldoon M, Teiler JP, Korber B, Shaw GM, Hahn BH Phenotypic properties of transmitted founder HIV-1. Proc Natl Acad Sci U S A. 2013;110(17):6626–33. PubMed PMID: . 23542380 10.1073/pnas.1304288110 PMC3637789

[14] Fenton-May AE, Dibben O, Emmerich T, Ding H, Pfafferott K, Aasa-Chapman MM, Pellegrino P, Williams I, Cohen MS, Gao F, Shaw GM, Hahn BH, Ochsenbauer C, Kappes JC, Borrow P Relative resistance of HIV-1 founder viruses to control by interferon-alpha. Retrovirology. 2013;10:146. PubMed PMID: . 24299076 10.1186/1742-4690-10-146 PMC3907080

[15] Veazey RS, Pilch-Cooper HA, Hope TJ, Alter G, Carias AM, Sips M, Wang X, Rodriguez B, Sieg SF, Reich A, Wilkinson P, Cameron MJ, Lederman MM Prevention of SHIV transmission by topical IFN-beta treatment. Mucosal Immunol. 2016. PubMed PMID: . doi: 10.1038/mi.2015.146 26838048 PMC4972705

[16] Tavel JA, Huang CY, Shen J, Metcalf JA, Dewar R, Shah A, Vasudevachari MB, Foll-mann DA, Herpin B, Davey RT, Polis MA, Kovacs J, Masur H, Lane HC Interferon-alpha produces significant decreases in HIV load. J Interferon Cytokine Res. 2010;30(7):461–4. PubMed PMID: . 20235638 10.1089/jir.2009.0090 PMC2964361

[17] Brook MG, Gor D, Forster SM, Harris W, Jeffries DJ, Tomas HC Suppression of HIV p24 antigen and induction of HIV anti-p24 antibody by alpha interferon in patients with chronic hepatitis B. Aids. 1988;2(5):391–3. PubMed PMID: . 3146270

[18] de Wit R, Schattenkerk JK, Boucher CA, Bakker PJ, Veenhof KH, Danner SA Clinical and virological effects of high-dose recombinant interferon-alpha in disseminated AIDS-related Kaposi's sarcoma. Lancet. 1988;2(8622):1214–7. PubMed PMID: . 2903953 10.1016/s0140-6736(88)90810-0

[19] Lane HC, Kovacs JA, Feinberg J, Herpin B, Davey V, Walker R, Deyton L, Metcalf JA, Baseler M, Salzman N, Manischewitz J, Quinnan G, Masur H, Fauci AS Anti-retroviral effects of interferon-alpha in AIDS-associated Kaposi's sarcoma. Lancet. 1988;2(8622):1218–22. PubMed PMID: . 2903954 10.1016/s0140-6736(88)90811-2

[20] Kovacs JA, Deyton L, Davey R, Falloon J, Zunich K, Lee D, Metcalf JA, Bigley JW, Sawyer LA, Zoon KC, Masur H, Fauci AS, Lane HC Combined zidovudine and interferon-alpha therapy in patients with Kaposi sarcoma and the acquired immunodeficiency syndrome (AIDS). Ann Intern Med. 1989;111(4):280–7. PubMed PMID: . 2757312 10.7326/0003-4819-111-4-280

[21] Lane HC, Davey V, Kovacs JA, Feinberg J, Metcalf JA, Herpin B, Walker R, Deyton L, Davey RT Jr., Falloon J, Polis MA, Salzman NP, Baseler M, Masur H, Fauci AS Interferon-alpha in patients with asymptomatic human immunodeficiency virus (HIV) infection. A randomized, placebo-controlled trial. Ann Intern Med. 1990;112(11):805–11. PubMed PMID: . 1971503 10.7326/0003-4819-112-11-805

[22] Krown SE, Gold JW, Niedzwiecki D, Bundow D, Flomenberg N, Gansbacher B, Brew BJ Interferon-alpha with zidovudine: safety, tolerance, and clinical and virologic effects in patients with Kaposi sarcoma associated with the acquired immunodefciency syndrome (AIDS). Ann Intern Med. 1990;112(11):812–21. PubMed PMID: . 1971504 10.7326/0003-4819-112-11-812

[23] Mildvan D, Bassiakos Y, Zucker ML, Hyslop N Jr., Krown SE, Sacks HS, Zachary J, Paredes J, Fessel WJ, Rhame F, Kramer F, Fischl MA, Poiesz B, Wood K, Ruprecht RM, Kim J, Grossberg SE, Kasdan P, Berge P, Marshak A, Pettinelli C Synergy, activity and tolerability of zidovudine and interferon-alpha in patients with symptomatic HIV-1 infection: AIDS Clincal Trial Group 068. Antivir Ter. 1996;1(2):77–88. PubMed PMID: . 11321183

[24] Krown SE, Aeppli D, Balfour HH Jr Phase II, randomized, open-label, community-based trial to compare the safety and activity of combination therapy with recombinant interferon-alpha2b and zidovudine versus zidovudine alone in patients with asymptomatic to mildly symptomatic HIV infection. HIV Protocol C91-253 Study Team. J Acquir Immune Defic Syndr Hum Retrovirol. 1999;20(3):245–54. PubMed PMID: . 10077172 10.1097/00042560-199903010-00005

[25] Azzoni L, Foulkes AS, Papasavvas E, Mexas AM, Lynn KM, Mounzer K, Tebas P, Jacobson JM, Frank I, Busch MP, Deeks SG, Carrington M, O'Doherty U, Kostman J, Montaner LJ Pegylated Interferon alfa-2a monotherapy results in suppression of HIV type 1 replication and decreased cell-associated HIV DNA integration. J Infect Dis. 2013;207(2):213–22. PubMed PMID: . 23105144 10.1093/infdis/jis663 PMC3532820

[26] Emilie D, Burgard M, Lascoux-Combe C, Laughlin M, Krzysiek R, Pignon C, Rudent A, Molina JM, Livrozet JM, Souala F, Chene G, Grangeot-Keros L, Galanaud P, Sereni D, Rouzioux C Early control of HIV replication in primary HIV-1 infection treated with antiretroviral drugs and pegylated IFN alpha: results from the Primoferon A (ANRS 086) Study. Aids. 2001;15(11):1435–7. PubMed PMID: . 11504966 10.1097/00002030-200107270-00014

[27] Manion M, Rodriguez B, Medvik K, Hardy G, Harding CV, Schooley RT, Pollard R, Asmuth D, Murphy R, Barker E, Brady KE, Landay A, Funderburg N, Sieg SF, Lederman MM Interferon-alpha administration enhances CD8+ T cell activation in HIV infection. PLoS One. 2012;7(1):e30306. PubMed PMID: . 22291932 10.1371/journal.pone.0030306 PMC3265460

[28] Lavender KJ, Gibbert K, Peterson KE, Van Dis E, Francois S, Woods T, Messer RJ, Gawanbacht A, Muller JA, Munch J, Phillips K, Race B, Harper MS, Guo K, Lee EJ, Trilling M, Hengel H, Piehler J, Verheyen J, Wilson CC, Santiago ML, Hasenkrug KJ, Dittmer U Interferon Alpha Subtype-Specific Suppression of HIV-1 Infection In Vivo. J Virol. 2016;90(13):6001–13. PubMed PMID: . doi: 10.1128/jvi.00451-16 27099312 PMC4907223

[29] Harper MS, Guo K, Gibbert K, Lee EJ, Dillon SM, Barrett BS, McCarter MD, Hasenkrug KJ, Dittmer U, Wilson CC, Santiago ML Interferon-alpha Subtypes in an Ex Vivo Model of Acute HIV-1 Infection: Expression, Potency and Effector Mechanisms. PLoS Pathog. 2015;11(11):e1005254. PubMed PMID: Pubmed Central PMCID: . doi: 10.1371/journal.ppat.1005254 26529416 PMC4631339

[30] Hunt PW, Martin JN, Sinclair E, Bredt B, Hagos E, Lampiris H, Deeks SG T cell activation is associated with lower CD4+ T cell gains in human immunodeficiency virus-infected patients with sustained viral suppression during antiretroviral therapy. J Infect Dis. 2003;187(10):1534–43. PubMed PMID: . 12721933 10.1086/374786

[31] Utay NS, Hunt PW Role of immune activation in progression to AIDS. Curr Opin HIV AIDS. 2016;11(2):131–7. PubMed PMID: Pubmed Central PMCID: . doi: 10.1097/coh.0000000000000242 26731430 PMC4750472

[32] Brenchley JM, Silvestri G, Douek DC Nonprogressive and progressive primate immunodefciency lentivirus infections. Immunity. 2010;32(6):737–42. PubMed PMID: Pubmed Central PMCID: . doi: 10.1016/j.immuni.2010.06.004 20620940 PMC2904340

[33] Fernandez S, Tanaskovic S, Helbig K, Rajasuriar R, Kramski M, Murray JM, Beard M, Purcell D, Lewin SR, Price P, French MA CD4+ T-cell deficiency in HIV patients responding to antiretroviral therapy is associated with increased expression of inter-feron-stimulated genes in CD4+ T cells. J Infect Dis. 2011;204(12):1927–35. PubMed PMID: . 22006994 10.1093/infdis/jir659

[34] Herbeuval JP, Boasso A, Grivel JC, Hardy AW, Anderson SA, Dolan MJ, Choug-net C, Lifson JD, Shearer GM TNF-related apoptosis-inducing ligand (TRAIL) in HIV-1-infected patients and its in vitro production by antigen-presenting cells. Blood. 2005;105(6):2458–64. PubMed PMID: . doi: 10.1182/blood-2004-08-3058 15585654

[35] Stoddart CA, Keir ME, McCune JM IFN-alpha-induced upregulation of CCR5 leads to expanded HIV tropism in vivo. PLoS Pathog. 2010;6(2):e1000766. PubMed PMID: Pubmed Central PMCID: . doi: 10.1371/journal.ppat.1000766 20174557 PMC2824759

[36] Li Q, Estes JD, Schlievert PM, Duan L, Brosnahan AJ, Southern PJ, Reilly CS, Peterson ML, Schultz-Darken N, Brunner KG, Nephew KR, Pambuccian S, Lifson JD, Carl-is JV, Haase AT Glycerol monolaurate prevents mucosal SIV transmission. Nature. 2009;458(7241):1034–8. PubMed PMID: . 19262509 10.1038/nature07831 PMC2785041

[37] Dion ML, Poulin JF, Bordi R, Sylvestre M, Corsini R, Kettaf N, Dalloul A, Boulassel MR, Debre P, Routy JP, Grossman Z, Sekaly RP, Cheynier R HIV infection rapidly induces and maintains a substantial suppression of thymocyte proliferation. Immunity. 2004;21(6):757–68. PubMed PMID: . 15589165 10.1016/j.immuni.2004.10.013

[38] Hardy GA, Sieg S, Rodriguez B, Anthony D, Asaad R, Jiang W, Mudd J, Schacker T, Funderburg NT, Pilch-Cooper HA, Debernardo R, Rabin RL, Lederman MM, Harding CV Interferon-alpha is the primary plasma type-I IFN in HIV-1 infection and correlates with immune activation and disease markers. PLoS One. 2013;8(2):e56527. PubMed PMID: . 23437155 10.1371/journal.pone.0056527 PMC3577907

[39] Massanella M, Tural C, Papagno L, Garcia E, Jou A, Bofill M, Autran B, Clotet B, Blanco J Changes in T-cell subsets in HIV-HCV-coinfected patients during pegylated interferon-alpha2a plus ribavirin treatment. Antivir Ter. 2010;15(3):333–42. PubMed PMID: . 20516553 10.3851/IMP1531

[40] Wilson EB, Yamada DH, Elsaesser H, Herskovitz J, Deng J, Cheng G, Aronow BJ, Karp CL, Brooks DG Blockade of chronic type I interferon signaling to control persistent LCMV infection. Science. 2013;340(6129):202–7. PubMed PMID: . 23580528 10.1126/science.1235208 PMC3704950

[41] Teijaro JR, Ng C, Lee AM, Sullivan BM, Sheehan KC, Welch M, Schreiber RD, de la Torre JC, Oldstone MB Persistent LCMV infection is controlled by blockade of type I interferon signaling. Science. 2013;340(6129):207–11. PubMed PMID: . 23580529 10.1126/science.1235214 PMC3640797

[42] Curtsinger JM, Valenzuela JO, Agarwal P, Lins D, Mescher MF Type I IFNs provide a third signal to CD8 T cells to stimulate clonal expansion and differentiation. J Immunol. 2005;174(8):4465–9. PubMed PMID: . 15814665 10.4049/jimmunol.174.8.4465

[43] Marshall HD, Urban SL, Welsh RM Virus-induced transient immune suppression and the inhibition of T cell proliferation by type I interferon. J Virol. 2011;85(12):5929–39. PubMed PMID: Pubmed Central PMCID: . doi: 10.1128/jvi.02516-10 21471240 PMC3126308

[44] McElrath MJ, Smythe K, Randolph-Habecker J, Melton KR, Goodpaster TA, Hughes SM, Mack M, Sato A, Diaz G, Steinbach G, Novak RM, Curlin M, Lord JD, Maenza J, Duerr A, Frahm N, Hladik F Comprehensive assessment of HIV target cells in the distal human gut suggests increasing HIV susceptibility toward the anus. J Acquir Immune Defic Syndr. 2013;63(3):263–71. PubMed PMID: . 23392465 10.1097/QAI.0b013e3182898392 PMC3683090

[45] Jacquelin B, Petitjean G, Kunkel D, Liovat AS, Jochems SP, Rogers KA, Ploquin MJ, Madec Y, Barre-Sinoussi F, Dereuddre-Bosquet N, Lebon P, Le Grand R, Villinger F, Muller-Trutwin M Innate immune responses and rapid control of infammation in African green monkeys treated or not with interferon-alpha during primary SIVagm infection. PLoS Pathog. 2014;10(7):e1004241. PubMed PMID: Pubmed Central PMCID: . doi: 10.1371/journal.ppat.1004241 24991927 PMC4081777

[46] Schellekens H, Niphuis H, Buijs L, Douw van der Krap P, Hochkeppel HK, Heeney JL Te effect of recombinant human interferon alpha B/D compared to interferon alpha 2b on SIV infection in rhesus macaques. Antiviral Res. 1996;32(1):1–8. PubMed PMID: . 8863990 10.1016/0166-3542(95)00955-8

[47] Fletcher CV, Staskus K, Wietgrefe SW, Rothenberger M, Reilly C, Chipman JG, Beil-man GJ, Khoruts A, Torkelson A, Schmidt TE, Anderson J, Perkey K, Stevenson M, Perelson AS, Douek DC, Haase AT, Schacker TW Persistent HIV-1 replication is associated with lower antiretroviral drug concentrations in lymphatic tissues. Proc Natl Acad Sci U S A. 2014;111(6):2307–12. PubMed PMID: Pubmed Central PMCID: . doi: 10.1073/pnas.1318249111 24469825 PMC3926074

[48] Johns TG, Kerry JA, Veitch BA, Mackay IR, Tutton PJ, Tymms MJ, Cheetham BF, Hertzog PJ, Linnane AW Pharmacokinetics, tissue distribution, and cell localization of [35S]methionine-labeled recombinant human and murine alpha interferons in mice. Cancer Res. 1990;50(15):4718–23. PubMed PMID: . 2369745

